# Comparative Study on Passive Film Formation Mechanism of Cast and PBF-LB/M-TC4 in Simulated Physiological Solution

**DOI:** 10.3390/ma17112583

**Published:** 2024-05-27

**Authors:** Ming Liu, Zhang Liu, Jie Wang, Yongqiang Zhang, Xin Gao

**Affiliations:** 1 Department of Materials Science and Engineering, Xi’an University of Technology, Xi’an 710048, China; 2 Shaanxi Province Key Laboratory of Corrosion and Protection, Xi’an University of Technology, Xi’an 710048, China; 3 Center for Advancing Materials Performance from the Nanoscale (CAMP-Nano), State Key Laboratory for Mechanical Behavior of Materials, Xi’an Jiaotong University, Xi’an 710049, China; zhangliu@stu.xjtu.edu.cn; 4 Shaanxi Zhou Doctor Dental Medical Co., Ltd., Xi’an 710086, China; 13379211721@163.com; 5 Xi’an QinTi Intelligent Manufacturing Technologies Co., Ltd., Xi’an 710061, China; zhyq613@mail.nwpu.edu.cn; 6 State Key Laboratory of Solidification Processing, Northwestern Polytechnical University, Xi’an 710072, China; 7 Research and Development Department, Beijing Med-Zenith Medical Scientific Corporation Limited, Beijing 101316, China

**Keywords:** laser powder bed fusion, TC4, passive film, X-ray photoelectron spectroscopy, Auger electron spectroscopy

## Abstract

Personalized laser powder bed fusion (PBF-LB/M) Ti-6Al-4V (TC4) has a broader application prospect than that of traditional casting. In this paper, the composition and corrosion resistance of the passive film formation mechanism of TC4 prepared by optimization of PBF-LB/M techniques and traditional casting were systematically studied in 0.9 wt.% NaCl at 37 °C by electrochemical technique and surface analysis. The rates of the passive film formation process, corrosion resistance and composition of TC4 show different characteristics for the different preparation processes. Although the rate of passive film formation of cast-TC4 was higher at the initial immersion, the open circuit potential was more positive, and the film thickness was larger after stabilization, those facts show no positive correlation with corrosion resistance. On the contrary, with no obvious defects on the optimized PBF-LB/M-TC4, the passive film resistance is 2.5 times more, the defect concentration is reduced by 30%, and the TiO_2_ content is higher than that of the cast-TC4, making the martensitic-based PBF-LB/M-TC4 exhibit excellent corrosion resistance. This also provides good technical support for the further clinical application of PBF-LB/M-TC4.

## 1. Introduction

Titanium (Ti) and its alloys have been widely employed in the medicine field. The studies on Ti and Ti alloys have already become the focus of study and the front field as biomedical implants [1,2,3], and they occupy “half of the country” in the field of medical metals. TC4 (Ti-6Al-4V) has a series of advantages, such as small density, high specific strength, excellent corrosion resistance, good toughness and weld ability, and has been resoundingly applied as artificial joints, vascular stents and orthopedic instruments [4,5,6].

As a kind of easy passivation alloy, the surface of Ti and Ti alloys can form a TiO_2_-containing passive film in the air protecting it from corrosion [7]. Nevertheless, the corrosion environment of Ti implants in human body is relatively severe, and the local acidizing and mechanical loading on implants may reduce the stability of its passive films, which may lead to further environmental pollution and damage of the formed film, affecting the durability of the implant. It has been confirmed that the corrosion resistance of TC4 in vivo or in vitro will be affected by many factors, such as the microstructure of materials, the coatings and the corrosive environment [8,9,10,11].

At present, the manufacturing process of TC4 includes casting, forging and machining [12,13], which are time-consuming and costly. With its complex operation, casting is at a high cost and causes environmental pollution, but it still is the conventional process for manufacturing oral prostheses. Hence, the design and manufacture of biomedical Ti alloys urgently require a fast and economical manufacturing method. In recent years, PBF-LB/M technology (also known as additive manufacturing) has unique advantages compared with traditional equal or reduced manufacturing. PBF-LB/M is a rapid prototyping technology for the direct manufacturing of terminal and near-terminal TC4 products [14,15]. The phase corrosion resistance in PBF-LB/M-TC4 is *β* > *α* > *α*’, and the V element in the *β* phase can stabilize the lattice structure, which is conducive to corrosion resistance; however, the V element in the *α*’ phase is relatively easy to dissolve, resulting in poor corrosion resistance [16,17,18,19]. There have also been plenty of studies on the passive film corrosion resistance of other Ti alloys [20,21,22,23]; a systematic and comprehensive evaluation of the corrosion behavior of Ti alloys is given, but it is worth noting that the good corrosion resistance of Ti and Ti alloys derives from the compact corrosion-resistant passive films.

In this study, the formation mechanism of the passive film and corrosion resistance of the optimized PBF-LB/M-TC4 prepared by PBF-LB/M and the traditional cast TC4 (cast-TC4) were systematically investigated in a simulated physiological solution (0.9 wt.% NaCl). Electrochemical techniques were applied to evaluate the passive film formation mechanism and corrosion resistance, and the composition and thickness of the passive film were further analyzed by surface technology.

## 2. Experimental Section

### 2.1. Material and Sample Preparation

The YLM-120 selective laser melting equipment produced by Jiangsu Yongnian laser forming Technology Co., Ltd. (Wuxi, Jiangsu, China) with high-precision circular working cylinder and multi-level precision guidance-sealing system was applied to fabricate the PBF-LB/M-TC4, which can effectively reduce the loss of metal powder and pollution. The raw material is 0.02 mm of spherical powder. In the preparation process, argon gas was selected as the protection gas. The following parameters could be selected based on our optimization: the hatch distance is 0.12 mm, the laser power is 275 W, the scanning velocity is 1100 mm/s, and the layer thickness is 30 μm. The comparison cast-TC4 sample was fabricated with lost wax.

The PBF-LB/M- and cast-TC4 cube samples (10 × 10 × 4 mm) were chosen for microstructure analysis, electrochemical testing and surface detection, respectively. For electrochemical testing samples, the one side (10 × 10 mm) was spliced with copper wire by conductive adhesive, the exposed testing area is 1 cm^2^, and the rest of the sample was sealed with epoxy resin. All samples were polished step by step with SiC sandpaper from 200–2000 # before the experiment, then washed with alcohol and deionized water and dried.

### 2.2. Electrochemical Tests

Electrochemical tests were performed on a VMP3 multi-channel electrochemical workstation (Biologic, Seyssinet-Pariset, France) with a standard three-electrode system. The reference electrode is SCE (the saturated calomel reference electrode), the platinum sheet (12 cm^2^) is the counter electrode, and the PBF-LB/M- and cast-TC4 sample is the working electrode. The simulated physiological solution with a mass fraction of 0.9 wt.% NaCl was applied in the test. Firstly, a long open circuit potential (OCP) was continuously monitored for 168 h. The polarization curves were measured after immersion for 0.5 h and 120 h, respectively. The scanning of the potentiodynamic polarization curve was from cathodic −250 mV vs. OCP to the anode with a 1 mV/s scanning rate and stopped when the anode current density exceeded 100 μA/cm^2^. Four potential ranges were applied for the cyclic voltammetry (CV) test: −2 V_SCE_ to 2.5 V_SCE_, −2 V_SCE_ to 2 V_SCE_, −1.5 V_SCE_ to 1.5 V_SCE_ and −1 V_SCE_ to 1 V_SCE_. The scanning started from cathode to anode and then back to the cathode, with five scanning cycles and a fixed scanning rate of 100 mV/s. The linear polarization was performed from cathode to anode with a scanning potential range of ±20 mV_SCE_ and a scanning rate of 0.2 mV/s. Electrochemical impedance spectroscopy (EIS) measurements were performed under OCP with a signal of 10 mV sine wave and a test frequency range of 100 kHz–10 mHz. ZsimpWin 3.5 software was applied to analyze the test results. The Mott–Schottky was tested with a fixed frequency and scanning rate of 1 kHz and 50 mV/s, and the potential range of scanning was −1.0 V_SCE_ to 3 V_SCE_. All electrochemical measurements were tested three times, and the representative results were given. The temperature for all electrochemical tests was controlled at 37 °C by a thermostat water bath.

### 2.3. X-ray Photoelectron Spectroscopy Analysis

The X-ray photoelectron spectroscopy (XPS) was applied (Thermo Scientific, Oxford, UK) to identify the passive film composition of PBF-LB/M- and cast-TC4 samples’ immersion in physiological solution for 168 h. The monochromator was Al Kα, the sensitivity was 100 kcps, the spectrum scanning range was 0–1350 eV, the wide scanning interval was 1 eV, the narrow scanning interval was 0.1 eV, and the spectrum was calibrated with C1s (285.0 eV). The composition of two TC4 passive films was analyzed by Xpspeak 4.1 software using the Gauss–Newton fitting mode.

### 2.4. Auger Electron Spectroscopy Analysis

Auger electron spectroscopy (AES) was performed on a PHI-700 (ULVAC-PHI, Chigasaki, Japan) equipped to analyze the passive films’ thickness variation of a two TC4 sample’s immersion in physiological solution for 168 h. A coaxial electron gun and CMA energy analyzer were adopted. Auger spectra were taken at 5 keV with an energy resolution of 0.1%, the incidence angle was 30°, and the vacuum of the analysis chamber was <3.9 × 10^−9^ Torr. The depth profile was obtained by etching a Φ100 nm spot on the surface of the passive film with Ar^+^ ions, and the thermal oxidation of standard SiO_2_/Si was adopted to determine the sputtering rate of 1 nm/min.

## 3. Results and Discussion

### 3.1. Microstructure Analysis

**Figure 1:**
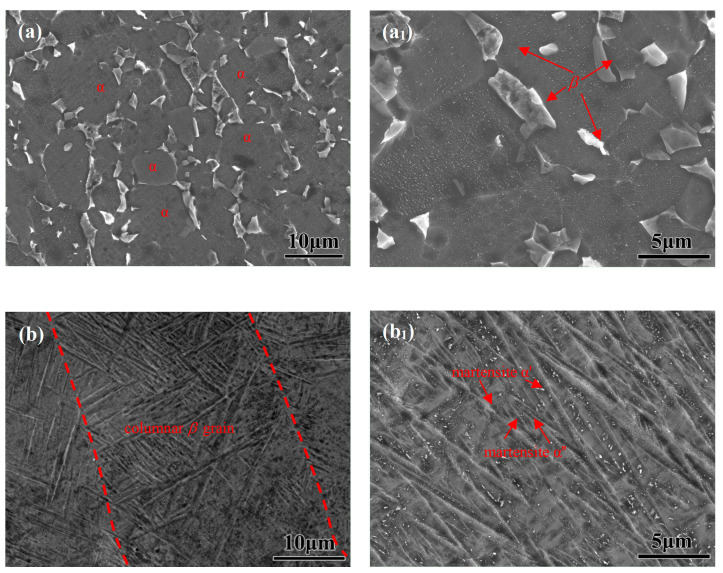
Scanning electron microscopy (SEM) of (**a**,**a_1_**) cast- and (**b**,**b_1_**) PBF-LB/M-TC4.

It can be seen from the scanning electron microscopy (SEM) in Figure 1a,a_1_ that the microstructure of traditional cast-TC4 is mainly equiaxed. The metastable *β* phase and the equiaxial *α* phase are uniformly distributed on the matrix, the duplex *α*-phase microstructure volume fraction is about 73.65%, the microstructure dispersion is high, and the grain size is about 9.75 μm. Figure 1b,b_1_ depicts the SEM morphology of PBF-LB/M-TC4.

The *β*-phase self-diffusion coefficient is higher, and the grain growth activation energy is lower, which leads to the epitaxial growth of grains. The martensitic lath in the *β*-phase columnar crystal has a preferred orientation, so the structure diagram shows an alternating phenomenon of light and dark. The longitudinal macro structure is an epitaxial growth columnar crystal with a length of up to a millimeter and a width of 27.34 μm. Upon further enlarging the longitudinal section of the metallographic structure, it is found that a large number of acicular martensite is distributed in the columnar crystal, which is basically parallel along the length direction and is composed of martensite *α*’ and martensite *α*″.

### 3.2. Electrochemical Analysis

#### 3.2.1. Open Circuit Potential

**Figure 2:**
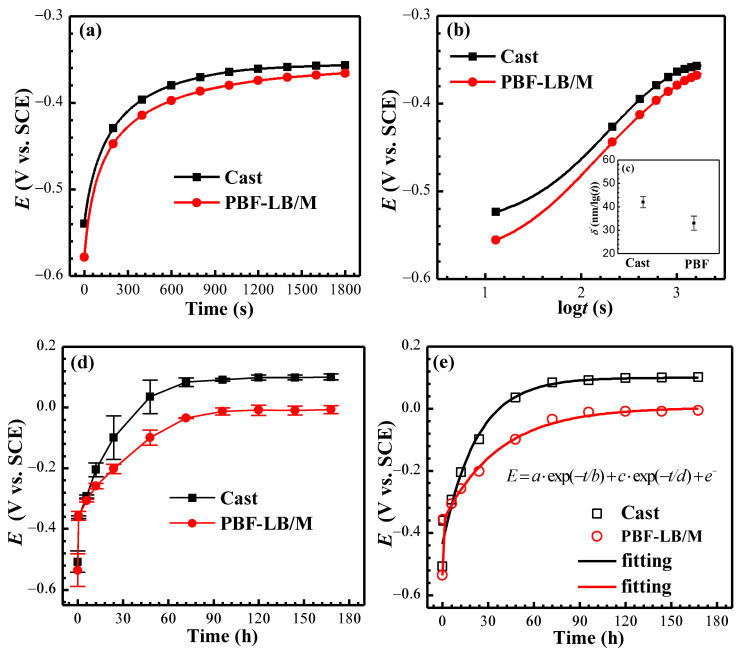
(**a**) The 1800 s OCPs of TC4 immersion in simulated physiological solution, (**b**) *E* vs. log*t* (s), (**c**) passive film formation rate, (**d**) OCP of 168 h, (**e**) fitting results of OCP.

Figure 2 depicts the OCPs of cast- and PBF-LB/M-TC4 after immersion in physiological solution for 1800 s.

The variation in OCP at the early stage of immersion can determine the passive film formation rate [24]. As can be seen from Figure 2a, the OCP of two TC4 increases in the positive direction rapidly. The passive film growth rate can be derived by Equation (1) [25]:

(1) $$E=\mathrm{const}.+2.303\;\delta^{-}/A\log t$$

wherein $\delta^{-}$ is the passivation film formation rate corresponding to log*t*. *A* can be calculated by Equation (2):

(2) $$A=\frac{nF}{RT}\alpha \delta \prime$$

where *α* and δ′ represent the charge transfer coefficient (α = 0.5) [26] and charge transfer process energy accumulation width (δ′ = 1), respectively. Numerous studies have demonstrated that TiO_2_ is the main composition in the passive film of Ti-related alloys [2,19], and the results of XPS and AES will support that the passive film of two TC4is mainly TiO_2_. Herein, the thickening of the passive film is assumed to be mainly through Ti^4+^ diffusion to the Ti and oxygen interface and *n* = 4 in Equation (2); the calculated *A* equals 78 nm/V. The early-stage formation rate of passive film δ′ can be derived (see Figure 2b) and shows the following order: Cast-TC4 > PBF-LB/M-TC4 (see Figure 2c).

The long-term OCP of the two TC4 continuous monitoring for 168 h in physiological solution is depicted in Figure 2d. The OCP increases rapidly at the initial 0.5 h, then rises slowly at 0.5–48 h and stabilizes at 72 h. The 168 h OCP of the cast- and PBF-LB/M-TC4 is 101 mV_SCE_ and −7 mV_SCE_, respectively. Here, the power function was applied to fitting the OCP vs. *t* [25]:

(3) $$E=a\cdot \exp(-t/b)+c\cdot \exp(-t/d)+e^{-}$$

where *a* − *e* are all constants. The *E* vs. *t* of two TC4 in 0.9 wt.% NaCl solution is fitted by Equation (3). It can be seen from the fitting results (see Figure 2e and Table 1) that the OCP of two TC4 alloy conforms well to the power function.

#### 3.2.2. Potentiodynamic Polarization

**Figure 3:**
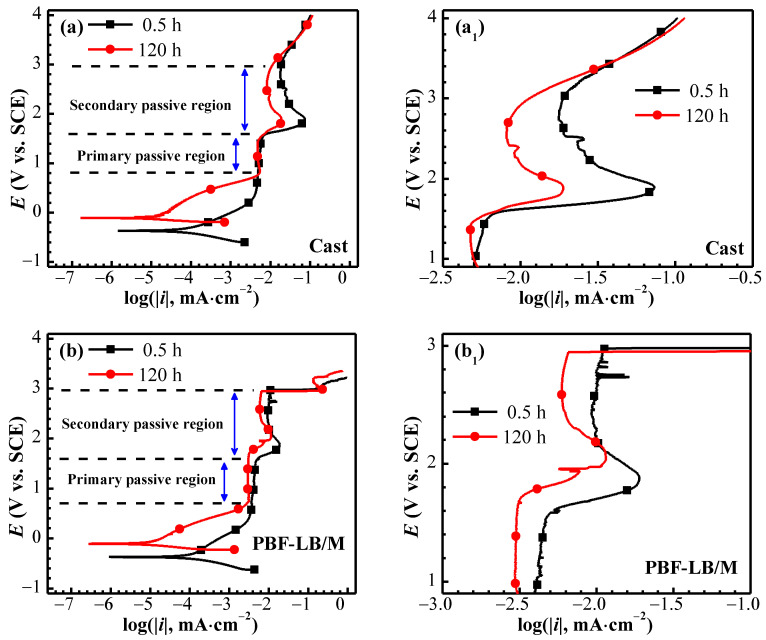
Potentiodynamic polarization curves of cast- and PBF-LB/M-TC4 immersed in physiological solution for 0.5 and 120 h. (**a**) cast-TC4, (**a_1_**) magnification of anode curve of cast-TC4, (**b**) PBF-LB/M-TC4 and (**b_1_**) magnification of anode curve of PBF-LB/M-TC4.

In terms of corrosion thermodynamics, a completely stabilized OCP of two TC4 needs at least 72 h of immersion (see Figure 2). Herein, the test of potentiodynamic polarization curves chosen for 0.5 h and 120 h corresponds to the OCP (see Figure 3).

The anode polarization curves exhibit typical metal passivation characteristics, and two obvious passivation zones can be observed without obvious activation to passivation transition. The primary and the secondary passivation regions are 0.8–1.6 V_SCE_ and 1.6–3 V_SCE_, respectively [27]. It can be seen from the fitting results derived from the potentiodynamic polarization curves shown in Table 2 that PBF-LB/M-TC4 has a relatively negative self-corrosion potential (*E*_corr_). The self-corrosion current density (*i*_corr_) from low to high at the first 0.5 h of immersion is as follows: cast-TC4 < PBF-LB/M-TC4 and inversely shows PBF-LB/M-TC4 < Cast-TC4 after 120 h of immersion. However, the maintaining passivity current density (*i*_pass_) of PBF-LB/M-TC4 is all along lower than that of cast-TC4 at fixed 1 V_SCE_.

The relatively limited integrity of the 0.5 h formed passive film may result in lower corrosion resistance. The metastable *β* phase and the equiaxial *α* phase of cast-TC4 promotes the passive film formation rate (Figure 3a). Nevertheless, the corrosion resistance of martensitic-based PBF-LB/M-TC4 (0.02 μA·cm^−2^) is higher than cast-TC4 (0.04 μA·cm^−2^) after immersion for 120 h and is positively correlated with the microstructure (Figure 1). Furthermore, the *i*_corr_ of two TC4 reduces greatly after 120 h of immersion, which is much lower than 0.1 μA·cm^−2^ [24], indicating that a corrosion-resistant passive film could be formed under a longer period of immersion.

#### 3.2.3. Cyclic Voltammetry

**Figure 4:**
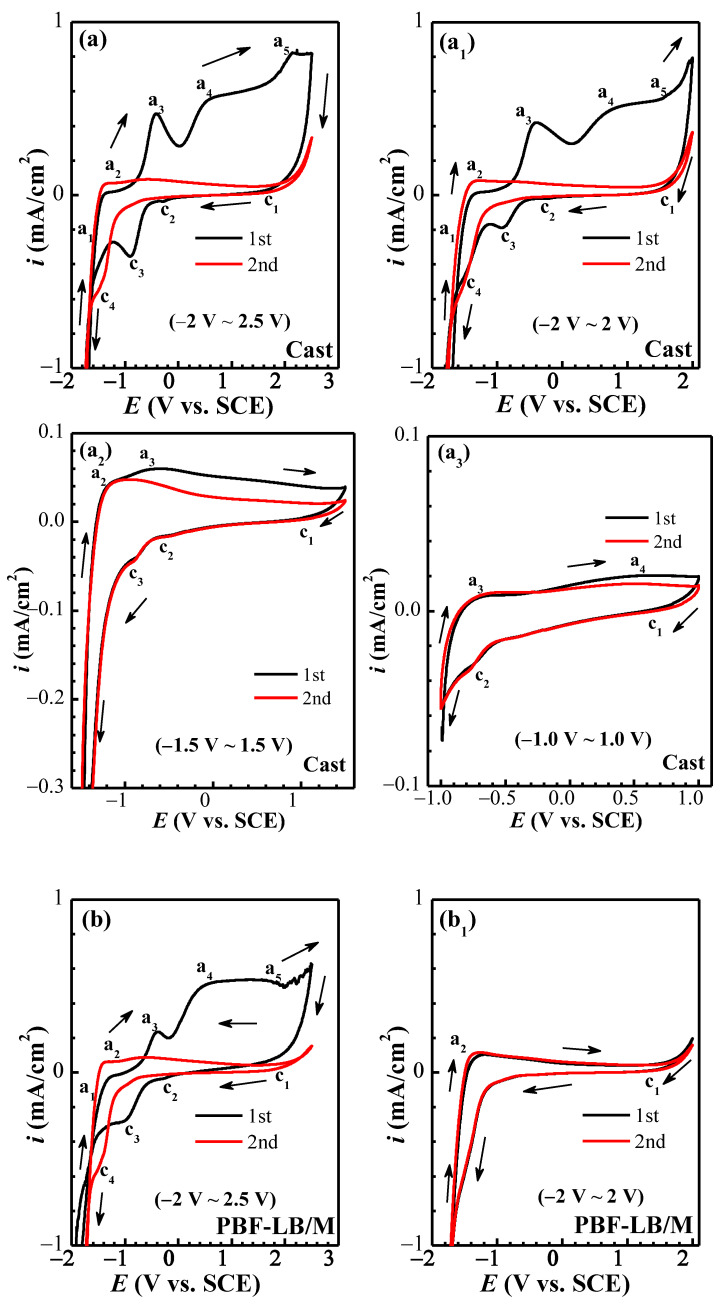

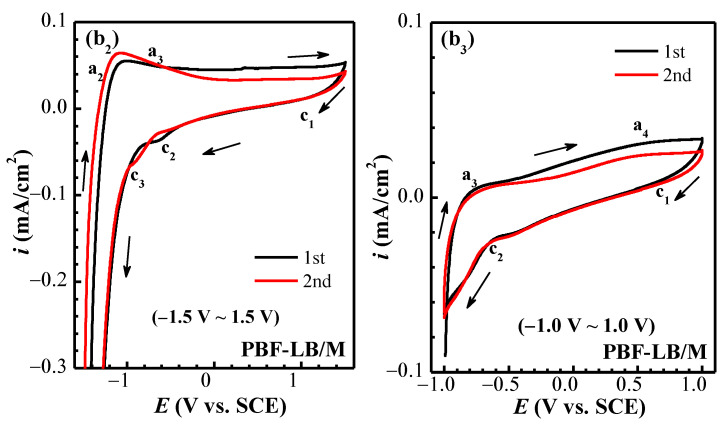
Cyclic voltammetry curves of TC4 with different scanning ranges of immersion in physiological solution: (**a**–**a3**) cast-TC4, (**b**–**b3**) PBF-LB/M-TC4.

The cyclic voltammetry (CV) curves of two TC4 tested in different potential ranges are exhibited in Figure 4. Four different scanning potential ranges—(−2.0–2.5 V_SCE_), (2.0–2.0 V_SCE_), (−1.5–1.5 V_SCE_) and (−1.0–1.0 V_SCE_)—were selected to distinguish redox reactions as much as possible. In the first cycle of the wide potential range (−2–2.5 V_SCE_) in Figure 4a,b, five distinct anode current peaks could be seen—a_1_ (−1.38 V_SCE_), a_2_ (−1.1 V_SCE_) and a_3_ (−0.59 V_SCE_)—corresponding to the oxidizing of Ti to Ti^2+^, Ti^2+^ to Ti^3+^ and Ti^3+^ to Ti^4+^, respectively [21,27]:

(4) $$a_{1}{:}_{\;}\mathrm{Ti}+H_{2}O↔\mathrm{TiO}+2H^{+}+2e^{-}$$

(5) $$a_{2}{:}_{\;}2\mathrm{TiO}+H_{2}O↔{\mathrm{Ti}}_{2}O_{3}+2H^{+}+2e^{-}$$

(6) $$a_{3}{:}_{\;}{\mathrm{Ti}}_{2}O_{3}+H_{2}O↔{2\mathrm{TiO}}_{2}+2H^{+}+2e^{-}$$

The other two peaks (a_4_ and a_5_) could be seen when forward scan potential exceeds 0.6 V_SCE,_ indicating that the electrode enters an oxygen-controlled zone.

Four cathodic current peaks can be detected in the reverse scanning, among which c_1_ (1.9 V_SCE_), c_2_ (−0.3 V_SCE_) and c_3_ (−0.87 V_SCE_) are the reduction peaks of a_4_ and a_5_, and a_3_ and a_2_, respectively. Due to the dissolved oxygen having a strong reduction reaction and reaching its limit at −0.6 V_SCE_ [28], part of the reduction process signal of Ti^4+^/Ti^2+^/Ti could be covered up, resulting in an insignificant reduction peak. The a_3_ peak is the highest and the c_2_ peak can be observed, indicating that the film is mainly Ti^4+^ [27]. The a_2_ peak is relatively small, and a larger c_3_ peak can be seen, indicating Ti^3+^ may be present in the passive film. Except for the relatively obvious a_2_ peak, the other anode peaks are sharply reduced in the second scanning cycle for two alloys, but a new reduction peak c_4_ (−1.4 V_SCE_) corresponding to the oxidation peak a_1_ can be observed, indicating that Ti^2+^ is in an unstable state.

For the test potential range of −2~2 V_SCE_ in Figure 4a_1_,b_1_, the CV curve of cast-TC4 is similar to that in Figure 4a.

Nevertheless, a stable film could be detected for PBF-LB/M-TC4, and only the inconspicuous peaks a_2_ and c_1_ could be seen on the overlapped CV curves. Only the a_2_ and a_3_ peaks and the corresponding c_2_ and c_3_ peaks can be seen in the scanning range of −1.5–1.5 V_SCE_ (see Figure 4a_2_,b_2_), indicating that Ti^4+^ and Ti^3+^ exist in the passive film. The c_3_ peak of cast-TC4 is smaller, suggesting that Ti^3+^ is unstable, while the c_2_ peak of PBF-LB/M-TC4 is relatively larger, implying that Ti^4+^ remains in the passive film and shows better corrosion resistance [24]. The a_3_, a_4_, c_1_ and c_2_ peaks can be seen while further shortening the potential range to −1–1 V_SCE_ (see Figure 4a_3_,b_3_), manifesting that TiO_2_ is mainly in the passive film.

The peaks of Al and V cannot be observed in the CV curves within the test potential ranges. Here, a preliminary judgement can be made that the two TC4 passive film is mainly Ti oxides, of which Ti^4+^ is the key component in the film. The stability of film from high to low is PBF-LB/M-TC4 > cast-TC4; the specific composition and change with thickness will be discussed in the XPS and AES section.

#### 3.2.4. Linear Polarization Curve

**Figure 5:**
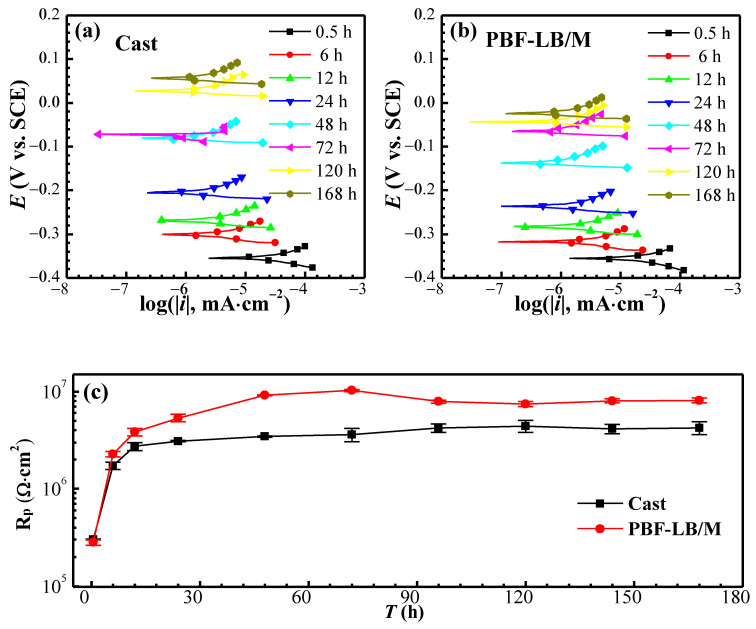
LPR curves of two TC4 immersed for different times: (**a**) cast-TC4, (**b**) PBF-LB/M-TC4 and (**c**) *R*_p_ fitting.

The linear polarization (LPR) curves and the fitting results after being immersed for different times are depicted in Figure 5.

As can be seen from Figure 5a,b, the *E*_corr_ moves upward, and the overall potential of the cast-TC4 is more positive than that of the PBF-LB/M-TC4 under the same conditions, which is consistent with the OCP part (see Figure 2c). The polarization resistance (*R*_p_) values after fitting the slope of the LPR curves is shown in Figure 5c. Despite the *E*_corr_ of the cast-TC4 being more positive than that of PBF-LB/M-TC4, the *R*_P_ shows the opposite trend except for immersion for 0.5 h. After 168 h of immersion, the *R*_P_ of PBF-LB/M-TC4 (8.2 × 10^6^ Ω·cm^2^) is twice that of cast-TC4 (4.1 × 10^6^ Ω·cm^2^), indicating that the passive film of PBF-LB/M-TC4 has excellent corrosion resistance.

#### 3.2.5. Electrochemical Impedance Spectroscopy

**Figure 6:**
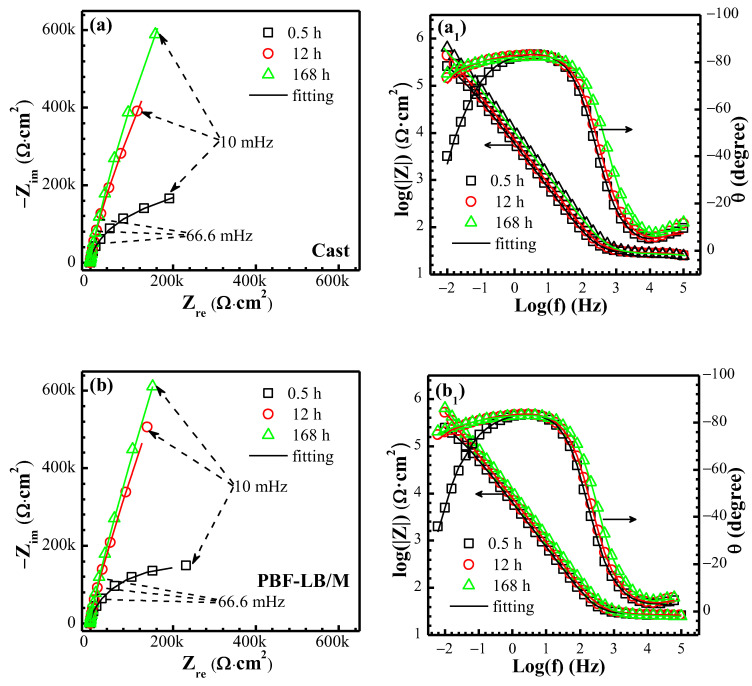
The EIS test results of two TC4 immersion in physiological solution: (**a**,**b**) Nyquist and (**a_1_**,**b_1_**) Bode.

The representative Nyquist (Figure 6a,b) and Bode (Figure 6a_1_,b_1_) plots of TC4 immersed in 0.9 wt.% NaCl solution are exhibited in Figure 6.

The radius of the capacitance loop in the Nyquist plot of two TC4 increases remarkably from 0.5 h to 168 h. The Bode plots of the phase angle of two TC4 show a wide arc of capacitance in the frequency range (10^3^–10^−2^ Hz), indicating that there are at least two superposed time constants and that after 12 h and 168 h of immersion, the phase angle gradually moves upward in the low-frequency region (10^0^–10^−2^ Hz). The impedance modulus |Z|_0.01_ is commonly applied to identify the corrosion resistance of the alloy [29]. The|Z|_0.01_ from high to low of the two alloys is PBF-LB/M-TC4 > cast-TC4 (see Figure 6), indicating that PBF-LB/M-TC4 has better corrosion resistance.

**Figure 7:**
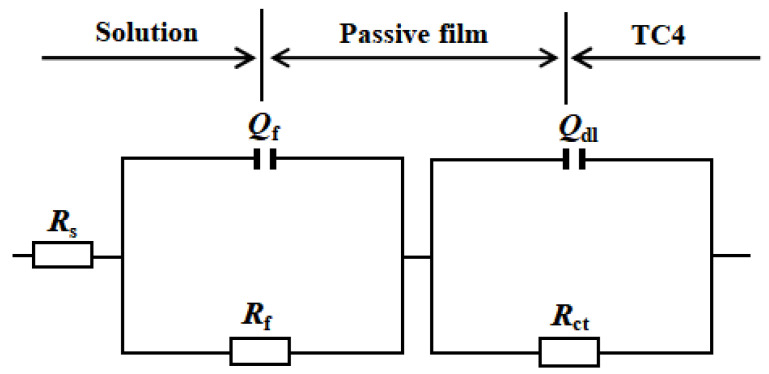
Equivalent circuit of TC4 immersion in physiological solution.

The equivalent circuit (EEC) in Figure 7 is often chosen to fit the passive film corrosion process of Ti and its alloys [9,27]. The model considers the passive film layer (*R*_f_, *Q*_f_) and in series the charge transfer layer (*R*_ct_, *Q*_dl_), where *R*_s_ is the solution resistance, *R*_f_ and *Q*_f_ are the passive film resistance and related double-layer constant phase element and *R*_ct_ and *Q*_dl_ are the charge transfer resistance and related electrical double-layer constant phase element, respectively. In general, capacitance is non-ideal due to the electrode surface roughness [27,28,29,30], which can be written as [30,31,32,33]

(7) $$Z_{Q_{\mathrm{dl}}}=\frac{1}{Y_{0}{\left(j\omega \right)}^{n}}$$

where *ω*, *j* and *n* are the imaginary unit, angular frequency and exponent, respectively, and the constant phase element is equal to the capacitance while *n* = 1 [27,28,29,30,31,32,33].

**Figure 8:**
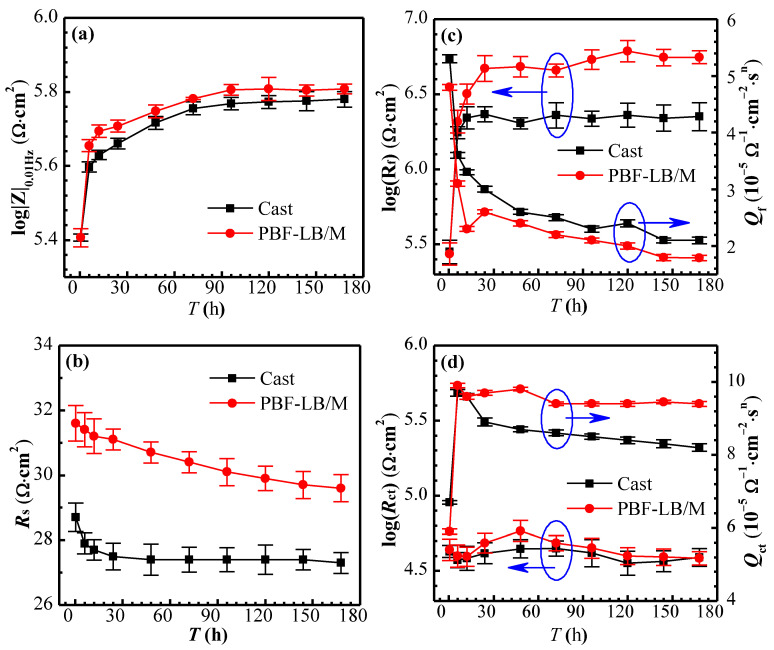
EIS fitting results of TC4 after immersion in physiological solution for different times. (**a**) |Z|_0.01_, (**b**) *R*_s_, (**c**) *R*_f_ and *Q*_f_, (**d**) *R*_ct_ and *Q*_ct_.

The EIS fitting results are shown in Figure 8. The *R*_s_ depicted in Figure 8b changes little in the range of 27–32 Ω·cm^2^ (cast-TC4 < PBF-LB/M-TC4).

The *R*_f_ of two TC4 increases rapidly from 10^5^ Ω·cm^2^ (0.5 h) to 10^6.3^ Ω·cm^2^ (12 h) and then fluctuates around 10^6.3^ Ω·cm^2^ for cast-TC4 (see Figure 8c). However, the *R*_f_ of the PBF-LB/M-TC4 continues to increase, reaching the order of 10^6.7^ Ω·cm^2^ after 24 h of immersion, and then fluctuates around this value in subsequent immersion. The *R*_ct_ depicted in Figure 8d of the two alloys is much smaller than that of the *R*_f_ and stabilizes at around 10^4.6^ Ω·cm^2^ during the whole test period, indicating that the resistance of the reaction mainly comes from the passive film, which is consistent with CV and LPR tests and indicates that the EEC model (Figure 7) applied here is appropriate. The *Q*_f_ and *R*_f_ show an apparent opposite trend (see Figure 8c): the passive film thickness of the alloy is usually preserved inversely proportionally to *Q*_f_ [27,30], and the sharply decreased *Q*_f_ with the extension of immersion time indicates a thickened passive film. Nevertheless, the *Q*_dl_ increases rapidly after immersion for 0.5 h, then reaches a maximum value at 6 h and then decreases slowly (see Figure 8d). Due to the relatively small and minimal difference in the *R*_ct_ of two TC4, herein the 168 h *R*_f_ was adopted to differentiate the corrosion resistance. PBF-LB/M-TC4 (10^6.7^ Ω·cm^2^) is 2.5 times more than that of cast-TC4 (10^6.3^ Ω·cm^2^), indicating that PBF-LB/M-TC4 has excellent corrosion resistance. In addition, it can be seen from the results [5,21] that the passive film resistance of the Ti alloy in simulated body fluids is also on the order of 10^6^ Ω·cm^2^, indicating that the test results here have high reliability and that further in vivo research is also needed.

#### 3.2.6. Mott–Schottky Analysis

**Figure 9:**
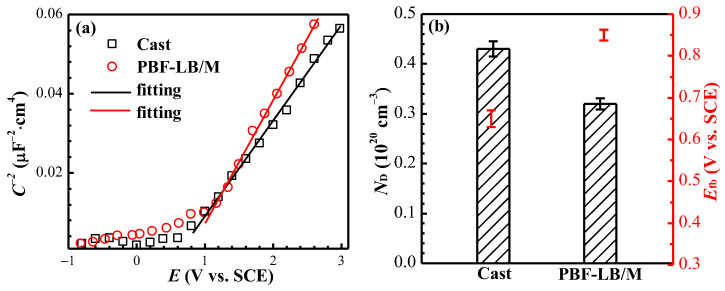
Mott–Schottky curves and fitting results of two TC4 immersed in physiological solution for 168 h: (**a**) M–S curve, (**b**) N_D_ and *E*_fb_.

The Mott–Schottky (M–S) curve after 168 h of immersion of two TC4 in physiological solution is exhibited in Figure 9.

The linear relation between 1/C^2^ and *E* (a) shown in Figure 9a can be described in Equation (8) [19,30]. The curves appear positive while the *E* is higher than the flat-band potential (*E*_fb_), the curves are all positive, indicating that the film of two TC4 shows an n-type semiconductor. The donor carrier density *N*_D_ can be derived from Equation (8) [19]:

(8) $$\frac{1}{C^{2}}=\frac{2}{{\varepsilon \varepsilon }_{0}eN_{D}}(E-E_{\mathrm{fb}}-\frac{kT}{e})$$

where, *C*, *ε*, *ε*_0_, *e*, *E*, *E*_fb_, *k* and *T* are the space charge layer capacitance, dielectric constant of passive film (for TiO_2_, ε = 100) [19,21], dielectric constant of vacuum (*ε*_0_ = 8.85 × 10^−12^ F/m), number of electrons (*e* = 1.602 × 10^−19^ C), applied potential, flat-band potential, Boltzmann constant (*k* = 1.38×10^−23^ J/K) and the thermodynamic temperature.

The *E*_fb_ and *N*_D_ fitting results in Figure 9b of PBF-LB/M- and cast-TC4 are 0.852, 0.653 V_SCE_ and 0.31, 0.43 (10^20^ cm^−3^), respectively. The defects of the passive film in cast-TC4 are 139% times those of PBF-LB/M-TC4, indicating that a more compacted passive film is formed on the PBF-LB/M-TC4 surface [19].

### 3.3. XPS Analysis

**Figure 10:**
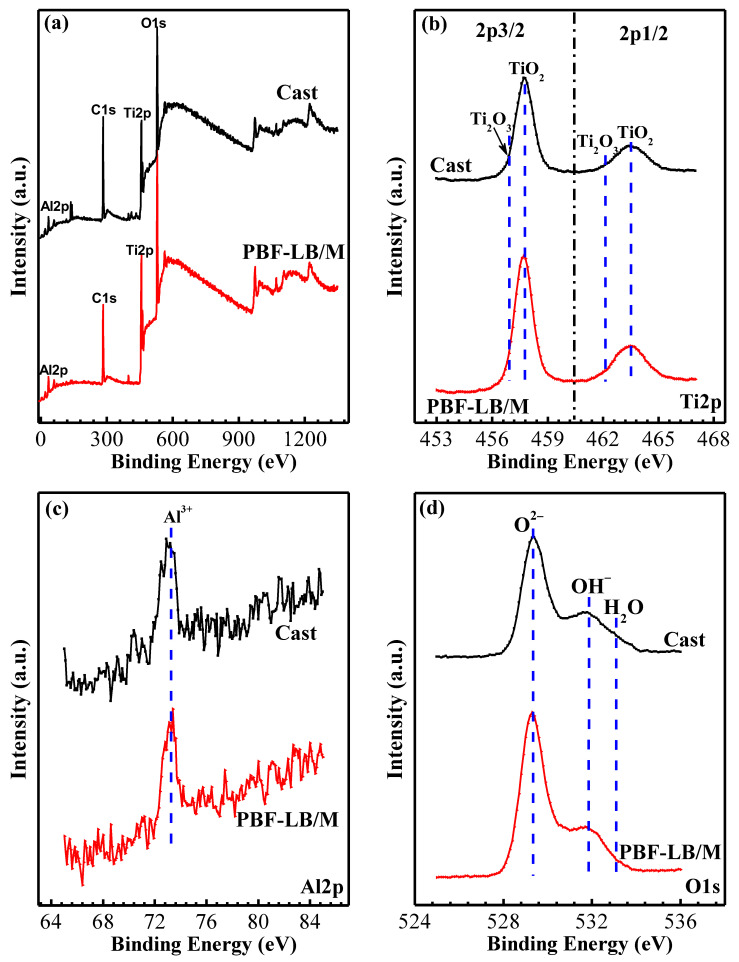
XPS peaks comparison of cast- and PBF-LB/M-TC4 immersed in physiological solution for 168 h. (**a**) full spectrum, (**b**) Ti 2p, (**c**) Al2p, (**d**) O1s.

Figure 10 exhibits the XPS peaks analysis of the two TC4 passive film immersed in physiological solution for 168 h.

For the full spectrum in Figure 10a, the film is mainly composed of Ti and O, weak Al, and hardly any V peak could be detected. Ti is mainly in TiO_2_ 2p3/2 (458.8 eV) and 2p1/2 (464.3 eV) in the film; small amounts of Ti_2_O_3_ 2p_3/2_ (456.8 eV) and 2p_1/2_ (462.0 eV) could also be detected (see Figure 10b). Our previous studies confirmed that Ti^4+^ mainly exists in the outer layer, and a low-priced Ti element mainly exists in the inner layer [7]. Only a weak Al peak can be detected (Figure 10c); the Al oxides are mainly Al(OH)_3_ (75.1 eV) and Al_2_O_3_ (74.3 eV). In Figure 10d, O is mainly composed of O^2−^ (530.2 eV); a small amount of OH^−^ (531.8 eV) and H_2_O (533 eV) can also be detected. The O^2−^ may be involved in the formation of Ti oxides (Ti_2_O_3_, TiO_2_) and Al oxides (Al_2_O_3_), and the OH^−^ mainly participates in the formation of Al(OH)_3_ and other compounds.

**Figure 11:**
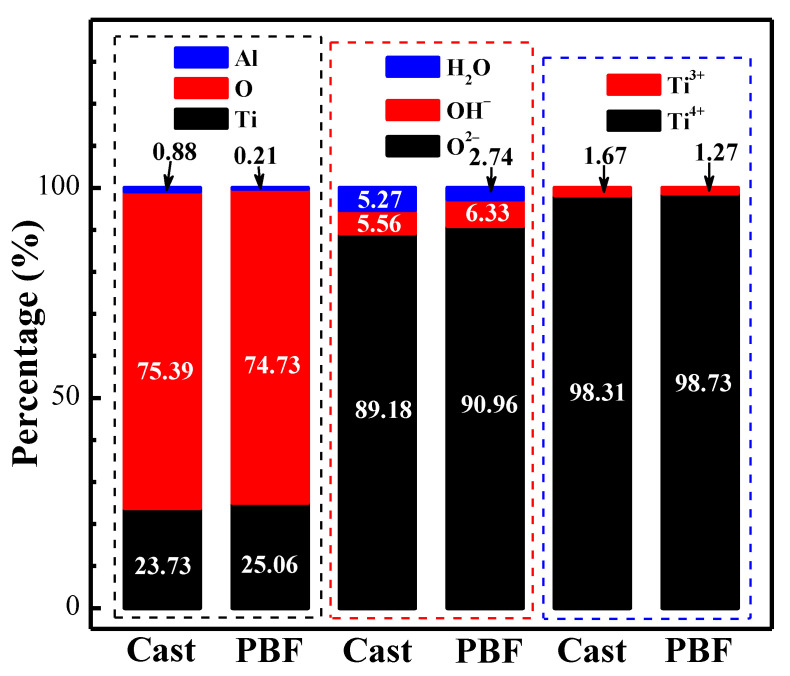
XPS element content comparison of passive film of cast- and PBF-LB/M-TC4 immersed in physiological solution for 168 h.

The valence states and contents of each major element of two TC4 alloys were summarized and analyzed, as shown in Figure 11.

In general, the O element accounts for the largest proportion (cast- and PBF-LB/M-TC4 are 75.39% and 74.73%, respectively), followed by Ti (cast- and PBF-LB/M-TC4 are 23.73% and 25.06%, respectively), and Al accounts for a small proportion, less than 1% (cast- and PBF-LB/M-TC4 are 0.88% and 0.21%, respectively). O and Ti mainly in the form of O^2−^ and Ti^4+^ conform to TiO_2_; the content of TiO_2_ in PBF-LB/M-TC4 is higher than that of cast-TC4, indicating that PBF-LB/M-TC4 has better corrosion resistance. These findings match perfectly with CV (Figure 4), LPR (Figure 5) and EIS (Figure 6) results.

### 3.4. AES Analysis

**Figure 12:**
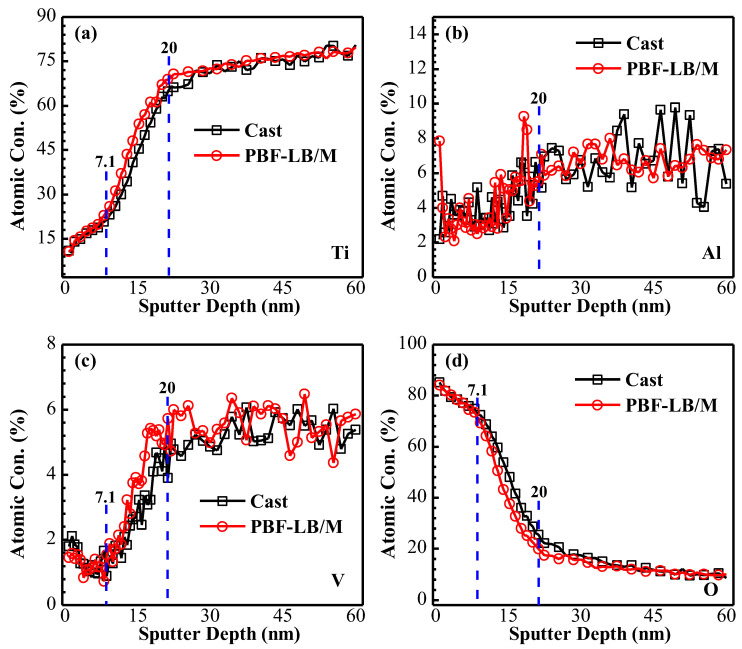
AES depth profile of cast- and PBF-LB/M-TC4 immersed in physiological solution for 168 h ((**a**)—**Ti,** (**b**)—Al, (**c**)—V, and (**d**)—O).

Figure 12 depicts the AES passive film depth profile of two TC4 immersed in physiological solution for 168 h.

In Figure 12a, Ti content increases slowly when the sputtering depth is in the region of 0–7.1 nm, increases sharply between 7.1 and 20 nm and changes little and becomes stable after the sputtering depth exceeds 20 nm. The overall Al content does not change much and stays at a relatively low level (Figure 12b). The V content decreases slowly in the region of 0–7.1 nm, increases between 7.1 and 20 nm and remains relatively stable after 20 nm (Figure 12c). The change trend of O content is completely opposite to that of Ti, which decreases in the range of 0–7.1 nm, increases between 7.1 and 20 nm and tends to be stable after sputtering exceeds 20 nm (Figure 12d). This again verifies that the outer passive film is mainly TiO_2_, while other low-priced Ti-oxide content increases in the inner layer, and the oxygen content decreases correspondingly. Despite the fact that the thickness of the PBF-LB/M-TC4 passive film (13.2 nm) is smaller than that of cast-TC4 (15.1 nm) (the location of the passive film thickness is defined as the oxygen content halved [34]), the passive film thickness is not a good criterion to evaluate corrosion resistance; on the contrary, the content of TiO_2_ in the passive film shows a positive correlation to corrosion resistance, with a higher concentration of TiO_2_ and fewer defects in the passive film enhancing better corrosion protection of martensitic based PBF-LB/M-TC4.

## 4. Conclusions

The formation and corrosion resistance of cast- and PBF-LB/M-TC4 passive films in physiological solution were studied by electrochemical techniques combined with surface analysis. The following main conclusions can be drawn:

1. The OCP of cast- and PBF-LB/M-TC4 conforms to the power function and increases rapidly with an extension of immersion time. Due to the large grain size of cast-TC4, the passive film formation rate shows the following order: cast-TC4 > PBF-LB/M-TC4.
2. The early-stage formed passive film shows a lower corrosion resistance. The metastable *β* phase and the equiaxial *α* phase of cast-TC4 promotes the passive film formation rate. A corrosion-resistant passive film could be formed during a longer period of immersion. The martensitic-based PBF-LB/M-TC4 shows better corrosion resistance than that of cast-TC4 after 120 h of immersion.
3. The *R*_P_ of LPR immersed for 168 h of PBF-LB/M-TC4 (8.2 × 10^6^ Ω·cm^2^) is twice of that cast-TC4 (4.1 × 10^6^ Ω·cm^2^), indicating the formed passive film of PBF-LB/M-TC4 has excellent corrosion resistance. The two TC4 passive film shows a typical n-type semiconductor, and the defect density in cast-TC4 is 139% times that of PBF-LB/M-TC4.
4. The passive film of two TC4 alloy is mainly Ti oxide. Ti^4+^ plays a dominant role, and the passive film’s stability from high to low is PBF-LB/M-TC4 > cast-TC4. Compared to the passive film thickness, the content of TiO_2_ in the passive film is a good criterion to evaluate corrosion resistance. A higher TiO_2_ concentration and fewer defects promote better corrosion protection of martensite-based PBF-LB/M-TC4.

## Figures and Tables

**Table 1:** OCP fitting results of two TC4 after continuous monitoring in physiological solution for 168 h.

Alloy	Fitting Result	*R* ^2^
Cast-TC4	E=−0.268⋅exp(−t/0.923)−0.268⋅exp(−t/0.923)+0.099	0.9666
PBF-LB/M-TC4	E=−0.369⋅exp(−t/1.528)−0.170⋅exp(−t/0.003)+0.004	0.9969

**Table 2:** Fitting values of potentiodynamic polarization parameters of cast- and PBF-LB/M-TC4.

Sample	Time (h)	*E*_corr_ (mV_SCE_)	*b*_c_, mV·dec^−1^	*i*_corr_, μA·cm^−2^	*i*_pass_*,* μA·cm^−2^
Cast-TC4	0.5	−383 ± 12	−144 ± 11	0.12 ± 0.03	6.5 ± 0.6
	120	−113 ± 8	−127 ± 9	0.04 ± 0.02	4.6 ± 0.4
PBF-LB/M-TC4	0.5	−385 ± 13	−191 ± 14	0.17 ± 0.03	4.4 ± 0.5
	120	−118 ± 7	−143 ± 6	0.02 ± 0.01	3.0 ± 0.3

## Data Availability

The original contributions presented in the study are included in the article, further inquiries can be directed to the corresponding author.

## Nomenclatures

PBF-LB/M	laser powder bed fusion of metals
TC4	Ti-6Al-4V
SCE	saturated calomel reference electrode
OCP	open circuit potential
CV	cyclic voltammetry
EIS	electrochemical impedance spectroscopy
M-S	Mott–Schottky
XPS	X-ray photoelectron spectroscopy
AES	Auger electron spectroscopy
SEM	scanning electron microscopy
$\delta^{-}$	passivation film formation rate
*α*	charge transfer coefficient
δ′	energy accumulation width
*E*_corr_	self-corrosion potential
*i*_corr_	self-corrosion current density
*i*_pass_	maintaining passivity current density
LPR	linear polarization
EEC	equivalent circuit
*R*_s_	solution resistance
*R*_f_	passive film resistance
*Q*_f_	passive film double-layer constant phase element
*R*_ct_	charge transfer resistance
*Q*_dl_	charge transfer double-layer constant phase element
*C*	space charge layer capacitance
*ε*	dielectric constant of passive film
*ε*_0_	dielectric constant of vacuum (*ε*_0_ = 8.85 × 10^−12^ F/m)
*e*	number of electrons (*e* = 1.602×10^−19^ *C*)
*E*	applied potential
*E*_fb_	flat-band potential
*k*	Boltzmann constant (*k* = 1.38 × 10^−23^ J/K)
*T*	thermodynamic temperature
